# Editorial: Breakthroughs in Cryo-EM with machine learning and artificial intelligence

**DOI:** 10.3389/fmolb.2025.1739481

**Published:** 2025-11-20

**Authors:** Edward T. Eng, Sonya M. Hanson, Ruben Sanchez-Garcia

**Affiliations:** 1 Simons Electron Microscopy Center, New York Structural Biology Center, New York, NY, United States; 2 Center for Computational Biology and Center for Computational Mathematics, Flatiron Institute, New York, NY, United States; 3 Department of Statistics, Mathematical, Physical and Life Sciences Division, University of Oxford, Oxford, United Kingdom; 4 School of Science and Technology, IE University, Madrid, Spain

**Keywords:** CryoEM, CryoET, machine learning, artificial intelligence, deep learning, computer vision, life sciences

Recent applications of machine learning (ML) and artificial intelligence (AI) in the biomedical science fields are transforming scientists’ approach to the structural sciences. Within cryogenic electron microscopy (cryo-EM), the development of new tools and the application of ML and AI workflows are revolutionizing data analysis, annotation, and interpretation. These methods are proving particularly effective at developing meaningful biological insights from information-rich, low-signal-to-noise datasets that are characteristic of cryo-EM experiments. Also, by automating labor-intensive tasks these approaches are accelerating both the pace and breadth of structural discoveries. This Research Topic on “Breakthroughs in Cryo-EM with Machine Learning and Artificial Intelligence” aims to highlight recent innovations that apply ML and AI toward understanding the molecular mechanisms of key biological processes through cryo-EM-driven research.

Towards this goal we had a variety of stories from Galaz-Montoya exploring the potential of AI-enhanced methods in structural histopathology to Matinyan et al. introducing novel approaches to reconstruct phase information from diffraction data. These contributions reflect a growing trend in cryo-EM to enhance data processing pipelines. In particular, Galaz-Montoya highlighted how cryo-EM, supported by AI-driven image analysis, could transform *in situ* visualization of cellular structures and even extend into clinical diagnostics by revealing nanoscale details of tissue and disease architecture. Meanwhile, Matinyan, et al. focused on a core technical challenge—retrieving lost phase information from single-molecule diffraction patterns—by developing a conditional generative adversarial network that bridges low-resolution image data with high-resolution diffraction patterns to reconstruct protein structures at atomic-level detail. Their model addresses a major bottleneck in computational phasing and offers an alternative to traditional single-particle approaches.

As new tools are developed and applied to different fields, it becomes increasingly important to create validation methods to assess results when these outputs may influence downstream data interpretation. Berkeley et al. highlights this issue by examining how ML-based density modification tools alter cryo-EM maps. Their findings reveal a more nuanced picture where these tools tend to enhance the overall structure of the macromolecules while unpredictably distorting ligand or ion densities, suggesting the need for caution in drug discovery contexts. One growing application of deep learning methods that Bansia et al. investigate is the automation of model building into cryo-EM maps and thereby helping researchers by accelerating tasks that were once manual and time-intensive. However, current models often struggle to generalize across different classes of macromolecules and resolution regimes, as available training sets are biased toward well-ordered, high-resolution structures. Moreover, cryo-EM frequently captures complexes in multiple conformational states, but many training sets may fail to capture this heterogeneity, thereby limiting the ability to accurately interpret dynamic and flexible regions of macromolecules.

With continual development and refinement of AI tools, Jeyaraj et al. comments on how cryo-EM workflows are now incorporating these approaches in conjunction with complementary biophysical approaches. The fields of cryo-EM and AI have their own metrics, but researchers are converging on common standards including depositing primary data, utilization of multiple validation and scoring methods, and clearly describing he protocols so others may replicate their results. This emphasis on reproducibility and reliability is echoed in Vargas et al.’s contribution, where the authors not only present a high-performing particle detection algorithm using a U-net-based semantic segmentation model but also share their training data and models publicly, promoting reproducibility and community-driven improvement. Finally, this Research Topic explores how ML can deepen our understanding of molecular behavior by enabling more interpretable representations of biological heterogeneity. Klindt et al. tackles the complex problem of latent space interpretability in cryo-EM developing a disentanglement method for the latent space of deep learning approaches used for heterogeneity analysis. This approach could help researchers to identify which latent dimensions correspond to meaningful conformational changes versus technical artifacts, enabling better interpretability of the dynamic processes that are central to protein function.

Overall, the works gathered in this Research Topic illustrate the accelerating integration of ML and AI into cryo-EM, transforming how structural data are processed, interpreted, and validated. Together, they highlight a shift from isolated methodological advances toward a more unified ecosystem in which computational intelligence complements experimental precision. This convergence is enabling cryo-EM to move beyond static snapshots of molecular structures toward richer, more dynamic representations of biological systems. As the field continues to evolve, careful attention to interpretability, reproducibility, and data sharing will be crucial to ensure that ML and AI-driven insights remain scientifically robust and biologically meaningful. We hope this Research Topic inspires further collaboration between computational and experimental communities as they continue to push the frontiers of molecular discovery.

